# Mechanisms involved in extraterritorial facial pain following cervical spinal nerve injury in rats

**DOI:** 10.1186/1744-8069-7-12

**Published:** 2011-02-10

**Authors:** Azusa Kobayashi, Masamichi Shinoda, Barry J Sessle, Kuniya Honda, Yoshiki Imamura, Suzuro Hitomi, Yoshiyuki Tsuboi, Akiko Okada-Ogawa, Koichi Iwata

**Affiliations:** 1 Department of Oral Diagnosis, Nihon University School of Dentistry, 1-8-13 Kandasurugadai, Chiyoda-ku, Tokyo, 101-8310, Japan; 2 Department of Physiology, Nihon University School of Dentistry, 1-8-13 Kandasurugadai, Chiyoda-ku, Tokyo, 101-8310, Japan; 3 Department of Oral biology, Faculty of Dentistry, University of Toronto, 124 Edward Street, Toronto, Ontario, M5G 1G6, Canada; 4 Division of Functional Morphology, Dental Research Center, Nihon University School of Dentistry, 1-8-13 Kandasurugadai, Chiyoda-ku, Tokyo 101-8310, Japan; 5 Department of Oral and Maxillofacial surgery, Nihon University School of Dentistry, 1-8-13 Kandasurugadai, Chiyoda-ku, Tokyo 101-8310, Japan; 6 Division of Applied System Neuroscience Advanced Medical Research Center, Nihon University Graduate School of Medical Science, 30-1 Ohyaguchi-Kamimachi Itabashi-ku, Tokyo 173-8610, Japan

## Abstract

**Background:**

The aim of this study is to clarify the neural mechanisms underlying orofacial pain abnormalities after cervical spinal nerve injury. Nocifensive behavior, phosphorylated extracellular signal-regulated kinase (pERK) expression and astroglial cell activation in the trigeminal spinal subnucleus caudalis (Vc) and upper cervical spinal dorsal horn (C1-C2) neurons were analyzed in rats with upper cervical spinal nerve transection (CNX).

**Results:**

The head withdrawal threshold to mechanical stimulation of the lateral facial skin and head withdrawal latency to heating of the lateral facial skin were significantly lower and shorter respectively in CNX rats compared to Sham rats. These nocifensive effects were apparent within 1 day after CNX and lasted for more than 21 days. The numbers of pERK-like immunoreactive (LI) cells in superficial laminae of Vc and C1-C2 were significantly larger in CNX rats compared to Sham rats following noxious and non-noxious mechanical or thermal stimulation of the lateral facial skin at day 7 after CNX. Two peaks of pERK-LI cells were observed in Vc and C1-C2 following mechanical and heat stimulation of the lateral face. The number of pERK-LI cells in C1-C2 was intensity-dependent and increased when the mechanical and heat stimulations of the face were increased. The decrements of head withdrawal latency to heat and head withdrawal threshold to mechanical stimulation were reversed during intrathecal (i.t.) administration of MAPK/ERK kinase 1/2 inhibitor PD98059. The area of activated astroglial cells was significantly higher in CNX rats (at day 7 after CNX). The heat and mechanical nocifensive behaviors were significantly depressed and the number of pERK-LI cells in Vc and C1-C2 following noxious and non-noxious mechanical stimulation of the face was also significantly decreased following i.t. administration of the astroglial inhibitor fluoroacetate.

**Conclusions:**

The present findings have demonstrated that mechanical allodynia and thermal hyperalgesia occur in the lateral facial skin after CNX and also suggest that ERK phosphorylation of Vc and C1-C2 neurons and astroglial cell activation are involved in orofacial extraterritorial pain following cervical nerve injury.

## Background

It has been reported that peripheral nerve injury causes marked neuronal excitability and gene expression in the central nervous system (CNS) as well as in the peripheral nervous system (PNS) [1,2]. Whole or partial peripheral nerve transection has been shown to generate a barrage of action potentials in the primary afferent neurons, including long lasting regular or burst firings, which are associated with the production of sensitization of nociceptive neurons in the CNS and/or PNS [3]. This central sensitization is associated with a variety of morphological and molecular changes in the CNS neurons and involved in neuroplastic changes in neural networks and synaptic transmission in the spinal cord dorsal horn (DH) and spinal trigeminal nucleus complex [1]. These neuroplastic changes in CNS nociceptive neurons caused by the peripheral nerve injury are thought to be significantly involved in pain abnormalities such as allodynia and hyperalgesia [4-6].

A number of studies have also reported that trigeminal nerve injury causes a marked hyperexcitability of trigeminal ganglion (TG) and trigeminal spinal subnucleus caudalis (Vc) neurons [7-10]. Allodynic and hyperalgesic nocifensive behaviors occur following mechanical and thermal stimulation of the whisker pad region innervated by the 2^nd ^(maxillary) branch of the trigeminal nerve 2 to 30 days after transection of the inferior alveolar nerve (IAN) which derived from the 3^rd ^(mandibular) branch [9]. Following IAN transection, Na^+ ^and K^+ ^channel activities, resting membrane-potential and hyperpolarization-activated current are changed in TG neurons innervated by the 2^nd ^branch of the trigeminal nerve, which are associated with an enhancement of TG neuronal excitability [9]. An enhancement of the Vc neuronal excitability reflecting central sensitization also occurs in rats with IAN transection. There is a significant increase in the background activity of Vc wide dynamic range (WDR) neurons in IAN-transected rats, and evoked responses following mechanical stimulation of the whisker pad area are also significantly larger in IAN-transected rats compared to Sham rats. These results indicate that IAN injury causes extensive changes in neuronal excitability in the uninjured territory of the orofacial region innervated by intact branches of the trigeminal nerve. The marked neuroplastic changes in Vc neurons are thought to be involved in the extraterritorial facial pain following IAN transection. Recently, non-neuronal cells such as glia have been reported to be involved in extraterritorial facial pain mechanisms in IAN-transected rats [11,12].

It has been reported that patients with cervical spinal nerve or muscle injury sometimes complain of pain abnormalities in the orofacial region as well as in the neck, and that local anesthesia of neck muscles sometimes reduces pain intensity in the orofacial regions as well as cervical muscle pain [13]. These report suggests that neck muscle or cervical spinal nerve injury may results in orofacial pain abnormalities. Some patients with temporomandibular joint pain may also show severe chronic pain in the neck region as well as orofacial region [14,15]. These various findings suggest that Vc and upper cervical spinal dorsal horn (C1-C2) nociceptive neurons interact with each other following cervical spinal nerve injury, resulting in severe chronic pain in the orofacial region. Convergence of cervical and trigeminal nerve afferents onto Vc and C1-C2 neurons is thought to be one of the possible mechanisms in orofacial pain abnormalities related to neck injury [16]. However, it is not fully understood how neck injury produces orofacial pain abnormalities. For these mechanisms, we hypothesized as follows: 1) the extracellular signal-regulated kinase (ERK) phosphorylation in Vc neurons is increased by mechanical stimulation to the lateral facial skin following cervical nerve injury, indicating that Vc and C1-C2 neurons are strongly activated and then Vc and C1-C2 neurons are also activated via interneurons. 2) the excitability of Vc and C1-C2 neurons is enhanced following cervical nerve injury via non-neuronal glial cells. These mechanisms involved may include increased excitability of Vc and C1-C2 neurons as reflected in ERK phosphorylation.

Therefore, we developed a cervical nerve injury model in the rats with C2-C4 nerve transection (CNX) and analyzed the nocifensive behavior, phosphorylated ERK (pERK) expression in Vc and C1-C2 neurons and astroglial cell activation with CNX.

## Results

### Nocifensive behavior to mechanical or heat stimulation of the lateral facial skin

**Figure 1:**
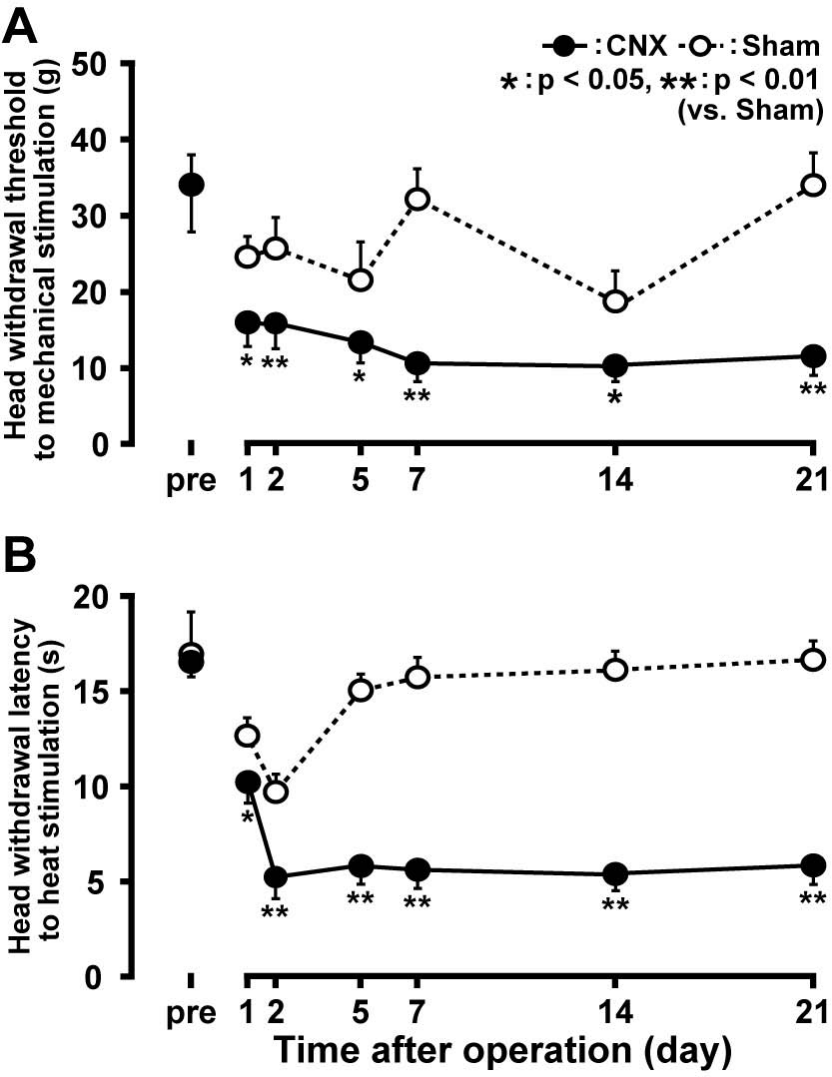
**Time-course change in head withdrawal threshold to mechanical stimulation of the lateral facial skin ipsilateral to CNX or Sham operation (A) and head withdrawal latency to heat stimulation of the lateral facial skin ipsilateral to CNX or Sham-operation (B) in CNX or Sham rats**. *: p < 0.05, **: p < 0.01 (vs. Sham).

Mechanical or heat stimulation was applied to the lateral facial skin ipsilateral to CNX before and 1-21 days after CNX. The head withdrawal threshold to mechanical stimulation of the lateral facial skin significantly decreased 1-21 days after CNX compared to Sham rats p *<*0.05, n = 7 in each group) (Figure 1A). The head withdrawal latency to heat stimulation of the lateral facial skin also decreased 1 days after CNX compared to Sham rats and the decrease in head withdrawal latency lasted through the experimental period (Figure 1B).

### pERK-LI cells in Vc and C1-C2 following mechanical or heat stimulation of the lateral facial skin

**Figure 2:**
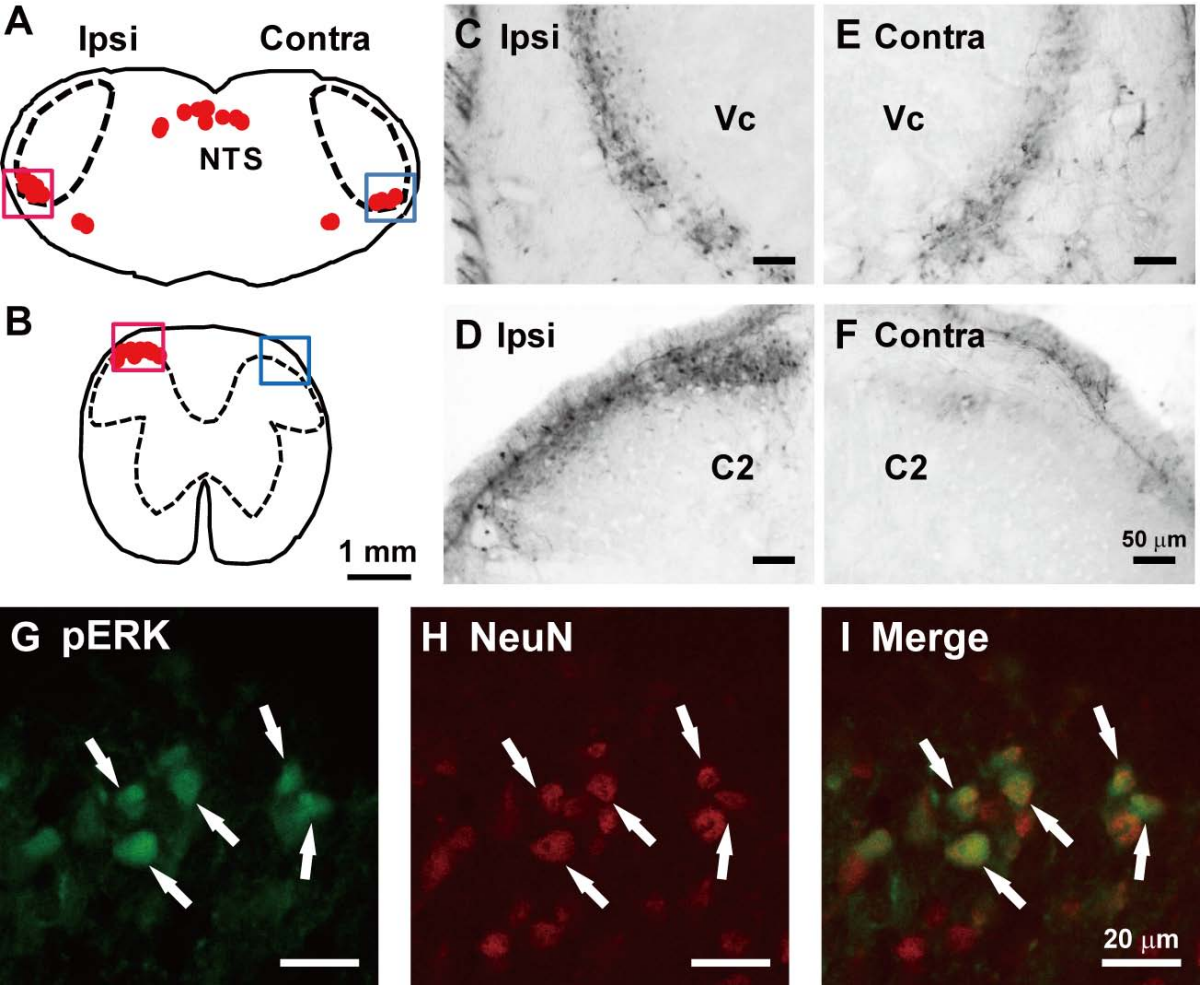
**Camera-lucida drawings (A: Vc, B: C2) and photographs (C-F) of pERK-LI cells in Vc (A, C and E) and C2 (B, D and F)**. C: Vc ipsilateral to CNX, D: C2 ipsilateral to CNX, E: Vc contralateral to CNX, F: C2 contralateral to CNX. G: pERK-LI cells in C2, H: NeuN-labeled cells in C2, I: merge G with H. Arrows indicate pERK and NeuN double labelled cells.

Many pERK-LI cells were observed in both sides of Vc after mechanical stimulation (60 g of the lateral face), and ipsilateral C1-C2 (from 2.0 to 6.0 mm caudal to the obex) following mechanical stimulation applied to the lateral facial skin ipsilateral to CNX (Figure 2A-D). pERK-LI cells were restricted in the ventral portion of the Vc, whereas those in the C1-C2 were segregated in the dorsal portion of the DH (Figure 2A-F). Most of pERK-LI cells were also restricted in the superficial laminae of the Vc and C1-C2. pERK-LI cells were double-stained with NeuN, indicating that pERK-LI cells expressed in Vc and C1-C2 were defined as neurons (Figure 2G-H). Since the number of pERK-LI cells was small in the Vc contralateral to CNX, we only analyzed the number of pERK-LI cells in the ipsilateral to CNX.

**Figure 3:**
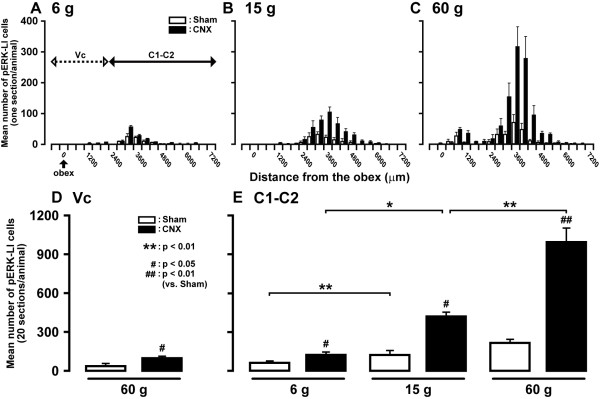
**Rostro-caudal distribution of pERK-LI cells in the Vc and C1-C2 following mechanical stimulation of the facial skin**. A: low-intensity (6 g), B: medium-intensity (15 g), C: high-intensity (60 g), D: mean number of pERK-LI cells in Vc induced by noxious mechanical stimulation (60 g) of the lateral facial skin in day 7 CNX and Sham rats, E: mean number of pERK-LI cells in C1-C2 induced by 6 g, 15 g or 60 g stimulation of the lateral facial skin in CNX or Sham rats. *: p < 0.05, **: P < 0.01 #: p < 0.05, ##: p < 0.01 (vs. Sham).

The rostro-caudal distribution patterns of the pERK-LI cells expressed by various mechanical stimuli were shown in Figure 3A-C. The distribution pattern of pERK-LI cells showed two peaks in Vc and C1-C2 approximately at 1.0 and 3.5 mm caudal to the obex, respectively. The pERK-LI cells were greatest in number at around 3.5 mm caudal to the obex at each stimulus intensity and the rostro-caudal distribution area of pERK-LI cells was graded in the C1-C2 following increase in the stimulus intensity. The number of pERK-LI cells in the C1-C2 increased from the lowest to the highest mechanical stimulus intensity. In the Vc, mean number of pERK-LI cells induced by noxious mechanical stimulation (60 g) was significantly larger in CNX rats than that of Sham rats (p < 0.05, n = 5 in each group) (Figure 3D). In C1-C2, mean number of pERK-LI cells induced by mechanical stimulation was significantly larger in CNX rats compared with that of Sham rats at each stimulus intensity and also showed intensity-dependency (6 g and 15 g: p < 0.05, 60 g: p < 0.01, n = 5 in each group) (Figure 3E).

**Figure 4:**
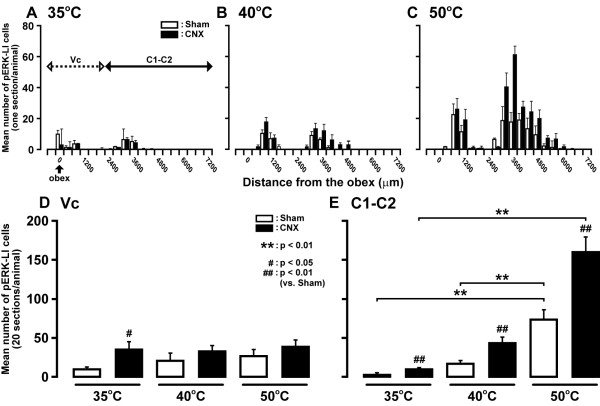
**Rostro-caudal distribution of pERK-LI cells in the Vc and C1-C2 following thermal stimulation of the facial skin**. A: low-intensity (35°C), B: medium-intensity (40°C), C: high-intensity (50°C), D: mean number of pERK-LI cells in Vc induced by thermal stimulation (35°C, 40°C and 50°C) of the lateral facial skin in day 7 CNX and Sham rats, E: mean number of pERK-LI cells in C1-C2 induced by 35°C, 40°C or 50°C stimulation of the lateral facial skin in CNX or Sham rats. **: P < 0.01, #: p < 0.05, ##: p < 0.01 (vs. Sham).

The rostro-caudal distribution patterns of the pERK-LI cells that were expressed by different intensities of thermal stimulation are shown in Figure 4A-C. The rostro-caudal distribution area of pERK-LI cells was obviously increased following increase in the thermal stimulus intensity. The distribution patterns of pERK-LI cells by thermal stimulation were similar to those following mechanical stimulation showing two peaks at around 1.0 and 3.5 mm caudal to the obex. The number of pERK-LI cells was greatest at around 3.5 mm caudal to the obex at each thermal stimulus and the number of them was significantly increased in CNX rats compared to Sham rats (p < 0.05, n = 5 in each group). In Vc, the mean number of pERK-LI cells induced by thermal stimulation (35°C) was significantly larger in CNX rats than that of Sham rats (p < 0.05, n = 5 in each group) (Figure 4D). On the other hand, we could not observe any significant differences in the number of pERK-LI cells in the Vc between CNX and Sham rats following 40°C and 50°C stimulation. The mean number of pERK-LI cells in CNX rats increased following increase in the thermal stimulus intensity (35, 40 and 50°C), and the mean number of pERK-LI cells in the C1-C2 was significantly larger in CNX rats compared with Sham rats at each stimulus intensity (p < 0.01, n = 5 in each group) (Figure 4E).

### Effect of MEK1/2 inhibitor PD98059 on ERK phosphorylation and nocifensive behavior

**Figure 5:**
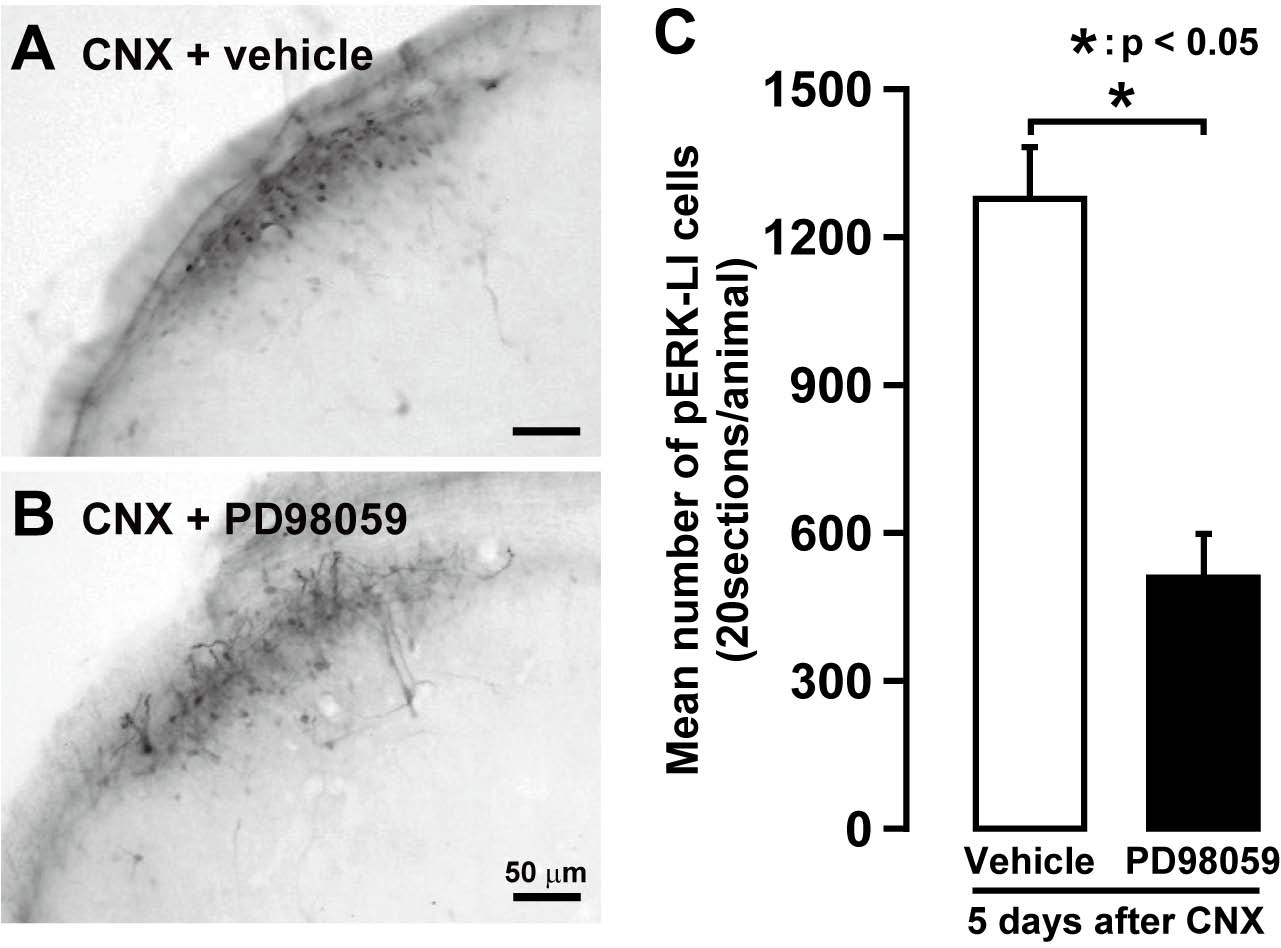
**Photograph of pERK-LI cells following mechanical (60 g) stimulation of the lateral facial skin in day 5 CNX rats with 5 days successive (A) i.t. administration of vehicle (saline) (A) or PD98059 (B)**. C: mean number of pERK-LI cells in Vc and C1-C2 following mechanical stimulation (60 g) of the lateral facial skin in CNX rats with vehicle or PD98059 i.t. administration. *: p < 0.05.

The effect of intrathecal (i.t.) administration of MEK1/2 inhibitor PD98059 (0.1 μg/μl, EMD Biosciences, La Jolla, CA) on ERK phosphorylation in Vc and C1-C2 neurons following noxious mechanical stimulation (60 g) of the lateral face was analyzed in CNX rats on day 5 after CNX (Figure 5). In CNX rats, a large number of pERK-LI cells was observed in the superficial laminae of the Vc in the CNX rats with i.t. vehicle injection, whereas a small number of pERK-LI cells was in the CNX rats with PD98059 administration (Figure 5A-B). The mean number of pERK-LI cells in Vc and C1-C2 was significantly smaller in CNX rats with PD98059 administration compared with vehicle-administrated rats (p < 0.05, n = 5 in each group) (Figure 5C).

**Figure 6:**
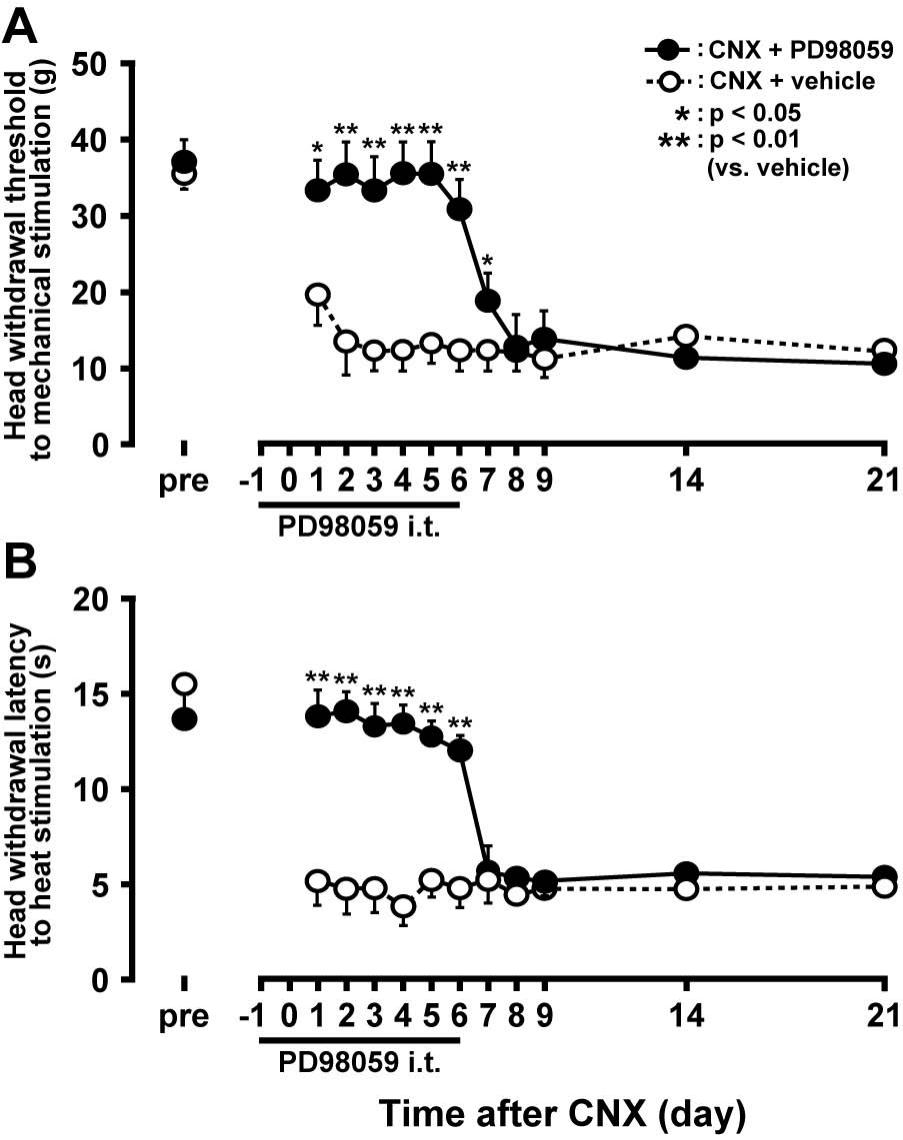
**Time-course change in head withdrawal threshold to mechanical stimulation (A) or head withdrawal latency to heat stimulation (B) of lateral facial skin with i.t. administration of PD98059 or vehicle in CNX rats**. *: p < 0.05, **: p < 0.01.

We also studied the effect of the PD98059 i.t. administration to nocifensive behavior induced by mechanical or heat stimulation (Figure 6). Both head withdrawal threshold to mechanical stimulation and head withdrawal latency to heat stimulation of the lateral facial skin were significantly lower and shortened in CNX rats with i.t. vehicle administration compared with those before CNX (n = 5 in each group). The lowering of the mechanical head withdrawal threshold and shortening of the heat-head withdrawal latency were attenuated by continuous PD98059 i.t. administration during 7-days administration period, and those nocifensive behaviours were observed one day after secession of the PD98059 administration.

### Astroglial activation and effect of astroglial cell inhibitor on nocifensive behavior, ERK and NMDAR1 (NR1) phosphorylation

**Figure 7:**
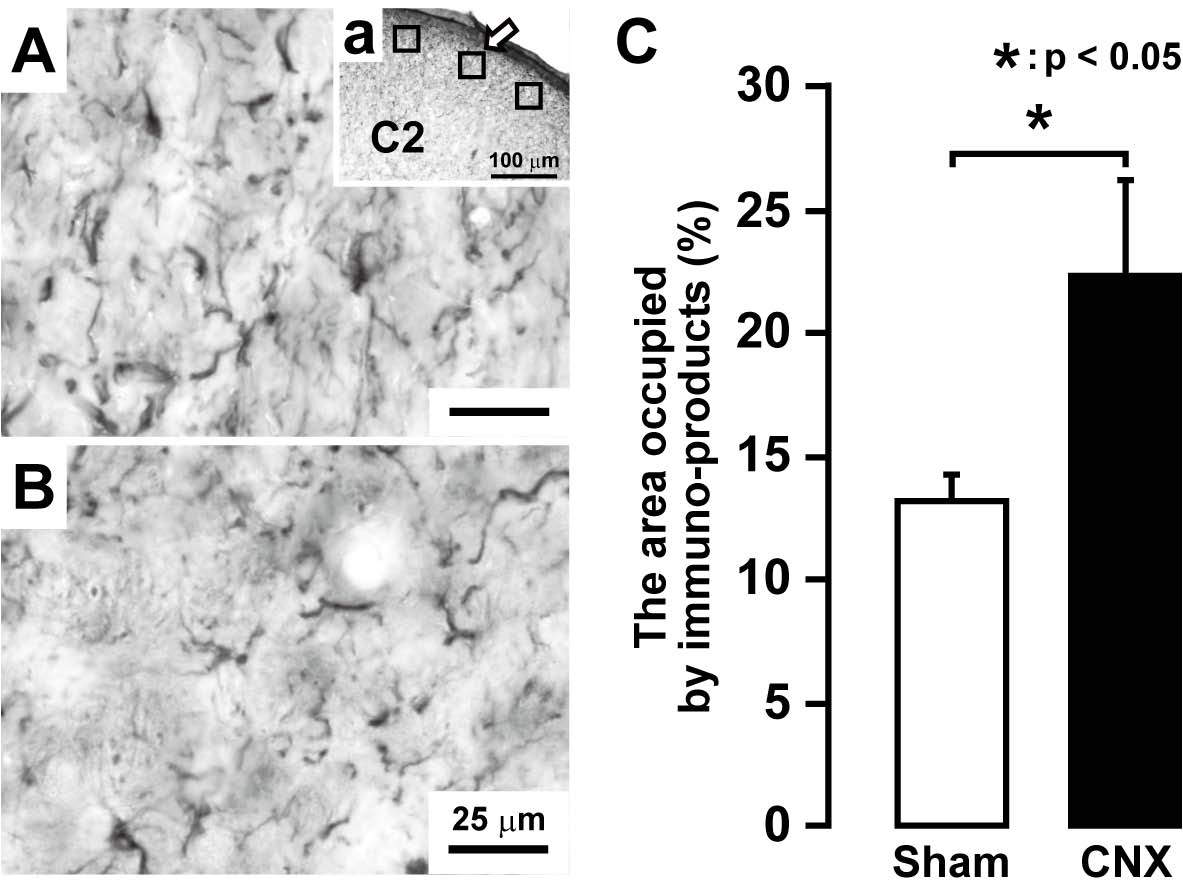
**GFAP-labeled cells in C2 in day 5 CNX (A) and Sham (B) rats**. C: mean percent area occupied by GFAP-labeled cells in C1-C2 on day 5 CNX and Sham rats. Aa: low magnification photomicrograph of C2 dorsal horn. Three square boxes were placed on the dorsal portion of C2. The low magnification photomicrograph of A corresponds to the area indicated by the arrow with box in Aa. *: p < 0.05.

**Figure 8:**
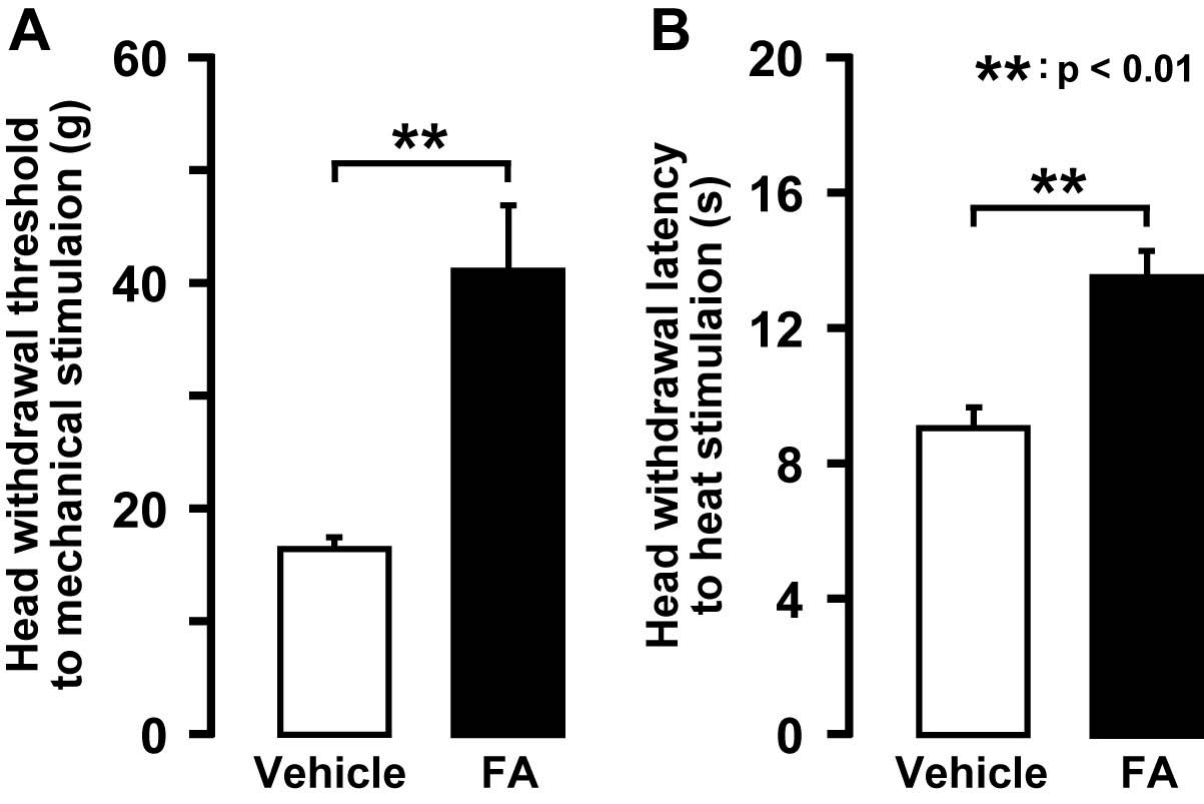
**The effect of 5 days successive i.t. vehicle or FA administration on nocifensive behavior in day 5 CNX rats**. A: head withdrawal threshold to mechanical stimulation of the lateral facial skin, B: head withdrawal latency to heat stimulation of lateral facial skin. **: p < 0.01.

**Figure 9:**
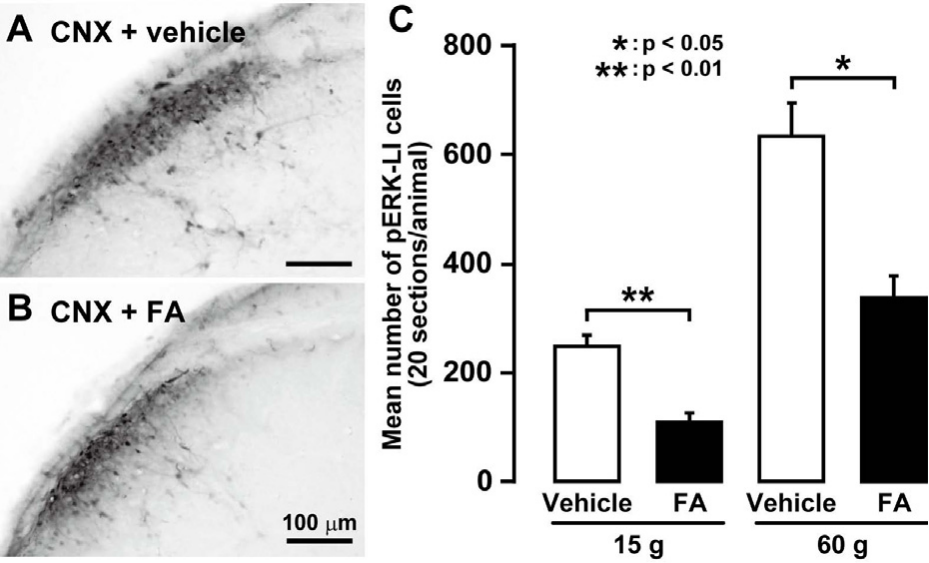
**The effect of 5 days successive i.t. vehicle or FA administration on pERK-LI cells in Vc and C1-C2 in CNX rats**. Photomicrographs of pERK cells following mechanical stimulation (60 g) of the lateral facial skin with i.t. administration of vehicle (A) and FA (B). C: mean number of pERK-LI cells following 15 g and 60 g mechanical stimulation of the lateral facial skin in CNX rats. *: p < 0.05, **: p < 0.01.

We studied astroglial cell activation in CNX rats as reflected by glial fibrillary acidic protein (GFAP) -labelled cells showing a large soma with thick processes in contrast to their morphological features in Sham rats (e.g. Figure 7A-B). The area occupied by the GFAP-labelled C1-C2 cells on day 7 after CNX operation was significantly larger in CNX rats than that in Sham rats (p < 0.05, n = 5 in each group) (Figure 7C). Many GFAP-labelled cells also showed glutamine synthetase (GS) immunoreactivity suggesting that GFAP-labelled (astroglial) cells were activated in CNX rats (Additional file 1: Figure S1A, B and C) but not in Sham rats (Additional file 1: Figure S1D, E and F). We also studied the effect of 5-days successive i.t. administration of the astroglial cell inhibitor FA or vehicle on nocifensive behavior to mechanical or thermal stimulation in CNX rats (Figure 8). Lowering of the mechanical head withdrawal threshold in CNX rats was significantly suppressed by i.t. FA administration compared with that of vehicle-injected rats (p < 0.01, n = 5 in each group) (Figure 8A). Moreover, the heat hyperalgesia in CNX rats was significantly suppressed by i.t. FA administration (p < 0.01, n = 5 in each group) (Figure 8B). When the effect of i.t. administration of FA on ERK phosphorylation in Vc and C1-C2 neurons was also analyzed in CNX rats, the number of pERK-LI cells induced by a non-noxious and noxious mechanical stimulation (15 and 60 g) in CNX rats was significantly suppressed by i.t. FA administration compared to vehicle-administrated rats (15 g: p < 0.01, 60 g: p < 0.05, n = 5 in each group) (Figure 9).

We studied the effect of 5 days successive i.t. administration of FA or vehicle on NR1 phosphorylation in CNX rats. NR1 phosphorylation was also inhibited by i.t. FA administration in the CNX rats suggesting that the deactivation of astroglial cells caused an inhibition of NMDA receptor activity (vehicle: additional file 1: Figure S1G, FA: additional file 1: FigureS1 H).

## Discussion

Primary afferent neurons are known to produce a barrage of action potentials following peripheral nerve injury [17]. Sodium channel accumulation in the transected nerve and the potassium channel down-regulation in the dorsal root ganglion (DRG) neurons are thought to be involved in the generation of high-frequency action potentials [18,19]. A variety of molecular changes are also produced in DH neurons as well as DRG neurons. Molecular changes in the DH and DRG neurons cause further increase in the neuronal excitability of the primary afferent neurons, resulting in sensitization of DH and DRG neurons [20]. Some previous clinical studies have reported that cervical muscle inflammation or cervical nerve injury causes a chronic pain in the orofacial region as well as neck pain [21]. Local anesthetic injection into cervical muscles can reduce the orofacial pain intensity as well as cervical muscle pain [13,22]. These data strongly suggest that cervical nerve injury produce extraterritorial facial pain. However, the underlying mechanisms of extraterritorial facial pain following cervical spinal nerve injury are unknown. For this reason, we developed a CNX model showing abnormal nocifensive behavior and performed the behavioral effects of astroglial cell modulator, and immunohistochemical analysis of pERK in Vc and C1-C2 neurons and astroglial cell activation in Vc and C1-C2.

### Relevancy of extraterritorial facial pain model

This is the first documentation of extraterritorial facial pain model caused by cervical nerve injury which is different from trigeminal neuropathic pain models caused by trigeminal nerve injury. The mechanism underlying CNX-induced extraterritorial facial pain may involve activation of C2-C4 spinal cord neurons followed by astroglial cell activation which might then affect the excitability of Vc and C1-C2 neurons. This mechanism is significantly different from trigeminal neuropathic pain models because of innervation areas, brain stem and spinal cord. Three branches of the trigeminal nerve locate next each other and innervate brain stem region, whereas C2-C4 nerves innervate the C1-C2 spinal cords. Thus the CNX model we have introduced in the present study more likely reflects a CNX-induced extraterritorial facial pain model rather than a trigeminal referred or neuropathic pain model.

Behavioral testing is important to define whether this is an appropriate model for extraterritorial facial pain [23]. Mechanical nocifensive behavior was tested for evidence of mechanical allodynia and heat nocifensive behavior was assessed for heat hyperalgesia [24]. We observed a significant reduction of the escape threshold to mechanical stimulation of the lateral facial skin in CNX rats compared to that of Sham rats. The head withdrawal latency to heat stimulation of the lateral facial skin was also significantly shorter in CNX rats compared to Sham rats. The reduced head withdrawal threshold to mechanical stimulation of the face and shortened head withdrawal latency to heating of the face lasted more than 3 weeks after CNX in this model. The changes in nocifensive behavior lasted for more than 40 days in some animals (data not shown). These suggest that mechanical allodynia and heat hyperalgesia are induced in the lateral facial skin following CNX in this rat model of cervical spinal nerve injury.

### Organization of Vc and C1-C2 nociceptive neurons

ERK in the DH neurons is one of the MAP kinase family that is phosphorylated after various types of noxious stimuli applied to the hind paw [25]. Strong noxious stimulation of peripheral tissues causes Ca^2+ ^influx into the nociceptive DH neurons via NMDA receptors, resulting in the activation of the neurons. The Ca^2+ ^influx further causes phosphorylation of the ERK in neurons [26]. In the trigeminal system, a large number of pERK-LI neurons have been found to be expressed in the Vc and C1-C2 regions within 5 min after capsaicin injection into various orofacial regions; these pERK-LI neurons were somatotopically organized in Vc and C1-C2, and the number of neurons showing such experiments was dependent on stimulus intensity [27,28]. These data strongly suggest that ERK phosphorylation in Vc and C1-C2 neurons is a reliable marker of excitable neurons following noxious stimulation of the orofacial region. In this study, we observed a large number of pERK-LI cells in the Vc bilaterally and C1-C2 in the side ipsilateral to the noxious stimulation of the lateral facial skin, indicating that pERK-LI cells in these regions are strongly activated by noxious stimulation of the facial skin. It has been reported that bilateral activation of nociceptive neurons in the Vc following noxious stimulation of the orofacial region is enhanced by vagal nerve transection and depressed by vagal nerve stimulation [29]. Together with these previous data, the present results suggest that Vc neurons are strongly activated after CNX and can be modulated by autonomic inputs, resulting in the bilateral activation of Vc neurons.

Another important observation was that the pERK-LI cell expression in the C1-C2 was intensity-dependent. The increase in the number of pERK-LI cells related to increases in the mechanical or heat stimulus intensity suggests that these neurons are involved in the encoding of the noxious stimulus intensity. The pERK-LI cells were also expressed in a restricted region in the dorsal portion of the ipsilateral C1-C2. Intensity-dependent changes in neuronal activity along with the somatotopic arrangement of nociceptive neurons are features involved in the sensory-discriminative aspect of pain [30,31]. These observations strongly support the view that C1-C2 nociceptive neurons are involved in sensory discrimination of extraterritorial facial pain following CNX.

We also observed that the i.t. administration of MEK1/2 inhibitor PD98059 caused significant suppression of the number of pERK-LI cells in Vc and C1-C2 compared to vehicle-administrated rats, and depressed the mechanical allodynia and heat hyperalgesia in CNX rats. These findings also suggest that the MAP kinase pathway is involved in enhancement of the excitability of Vc and C1-C2 neurons following CNX. However, it has also been reported that ERK phosphorylation occur in activated astroglial cells [32]. Thus, we could not exclude the possibility that PD98059 might affect astroglial cell activation following i.t. administration as well as neuronal excitability.

### Possible mechanisms of Vc and C1-C2 neuronal hyperactivation

It has been reported that not only Vc neurons but also C1-C2 neurons receive noxious inputs from the orofacial region [33,34]. These neurons are classified as WDR neurons and NS neurons. WDR neurons are responded to noxious as well as non-noxious stimuli. On the other hand, nociceptive specific (NS) neurons are exclusively responded to noxious stimuli. C1-C2 nociceptive neurons receiving orofacial regions are characterized by the large receptive field receiving noxious inputs from a wide area of the orofacial region [16]. WDR and NS neurons in Vc are known to be sensitized following peripheral nerve injury or inflammation in the orofacial region [35,36]. Sensitization of these neurons causes a barrage of action potentials conveyed to the higher CNS regions involving in the sensitization of thalamic and cortical nociceptive neurons [37,38]. Although neuronal excitability was not tested in this study, we observed significant increases in the number of pERK-LI cells in the Vc and C1-C2 regions in CNX rats. Taken together, this findings suggest that nociceptive information is conveyed to the higher CNS regions by sensitized WDR and NS neurons in Vc and C1-C2 following cervical spinal nerve injury, resulting in extraterritorial facial pain.

It has recently been reported that activated astroglial cells in the DH following peripheral nerve injury are involved in enhancement of the synaptic transmission in the CNS [39]. In the trigeminal system, Piao et al. have reported astroglial cell activation in Vc following trigeminal nerve injury [12]. Okada-Ogawa et al. have also reported that activated astroglial cells are expressed in the Vc at day 7 after IAN transection, and that i.t. injection of the astroglial cell inhibitor FA causes strong depression of Vc nociceptive neuron excitability in IAN-transected rats [11]. We also observed a large number of activated astroglial cells in Vc and C1-C2 at day 7 after CNX. Many GFAP-labeled cells also showed GS immunoreactivity suggesting that GFAP-labeled (astroglial) cells were activated in the CNX rats. The i.t. administration of FA also produced significant decrease in the nocifensive behavior in CNX rats at day 5 after cervical spinal nerve injury. Furthermore, we observed obvious decrease in NR1 phosphorylation in CNX rats. Together with the previous data, the present results suggest that astroglial cells are also involved in the sensitization of Vc and C1-C2 nociceptive neurons in CNX rats.

We counted the number of pERK-LI cells and measured the density of GFAP immunostaining to evaluate the activation of neuron and glial cells in the Vc and C1-C2. However, these do not indicate direct evidences for the activation of neurons and glial cells. Although it is highly possible that ERK phosphorylation in Vc and C1-C2 neurons and enlargement of the areas occupied by GFAP immuno-products indicate the activation of neurons and astroglial cells in the Vc and C1-C2, there are some limitations to interpret neuronal and glial cell activation in the Vc and C1-C2 from the present study.

## Conclusions

The novel extraterritorial facial pain model developed by cervical spinal nerve transection in rats manifested a large number of pERK-LI cells expressed in the Vc and C1-C2 as well as enhanced nocifensive behavior and both pERK expression and nocifensive behavior in CNX rats could be depressed by i.t. administration of PD98059. We also observed increased number of activated astroglial cells in the Vc and C1-C2 in CNX rats. The i.t. administration of the astroglial inhibitor FA also significantly depressed the pERK expression and enhanced nocifensive behavior in CNX rats. These findings suggest that astroglial cells in Vc and C1-C2 are strongly activated after the cervical spinal nerve injury, and their activation may be involved in the enhancement of Vc and C1-C2 neuronal excitability that involves ERK phosphorylation in the sensitized neurons, resulting in extraterritorial facial pain after cervical nerve injury.

## Methods

The present experiment was conducted under blind conditions. The experimenters who prepared the CNX model, measured the nocifensive behavior and conducted immunohistochemical staining were different, and the latter person was not aware of the rat's condition (experimental or control).

### Animals

Adult male Sprague Dawley rats (200-300 g) were used in this study (Japan SLC, Shizuoka, Japan). Rats were maintained in a climate-controlled room on a 12 h light/dark cycle (lights on at 07:00) with food and water available *ad libitum*. Every effort was made to minimize the number of animals used and their suffering. The experiments were approved by the Animal Experimentation Committee at Nihon University School of Dentistry, and procedures were performed according to the guidelines of the International Association for the Study of Pain [40].

### C2-C4 spinal nerve transection

Rats were initially anesthetized with sodium pentobarbital intraperioneally (i.p.; 50 mg/kg, Kyoritsu, Tokyo, Japan) and were placed on a warm mat (37°C). An incision was made on the neck skin and C2-C4 spinal nerves were exposed through the trapezius muscles. The C2-C4 spinal nerves were tightly ligated at two points of the nerve trunk and transected in the middle of two ligations, and then trapezius muscles and neck skin were sutured with 5-0 silk. For the Sham rats, the trapezius muscles and the neck skin were cut and sutured without nerve transection. After surgery, benzyl penicillin potassium (20,000 units, Penicillin G potassium, Meiji Seika, Tokyo, Japan) was administrated intramuscularly to prevent infection.

### Behavioral testing

First, rats were trained daily to stay in a plastic cage for 20 min, to protrude their perioral region including the lateral facial skin through a hole on the wall of the plastic cage for 5-10 min and to keep their snout protruding through a hole on the wall while mechanical or heat stimulation was applied to lateral facial skin [41]. Rats could escape freely from stimulations under this condition. The mechanical stimulation was applied daily to the lateral facial skin ipsilateral to CNX with von Frey filaments on each of 21 days after CNX. The head withdrawal threshold to mechanical stimulation was defined as the minimum pressure needed to evoke an escape more than 3 times of 5 stimuli. Similarly, heat stimulation was applied to the lateral facial skin by using a radiant heat stimulator; the head withdrawal latency to heat stimulation was measured.

### Effect of PD98059 or FA on nocifensive behavior

Under sodium pentobarbital anesthesia (50 mg/kg, i.p.), a laminectomy was performed at the L5 spinal cord and the dura was opened before CNX or sham operation. A microsilicon tube was inserted beneath the dura mater until the tip of the tube could reach the C3-C5 spinal cord. A microsilicon tube was connected to a mini-osmotic pump (Alzet model 2001, Cupertino, CA), which was filled with mitogen-activated protein kinase (MAPK) kinase 1/2 (MEK1/2) inhibitor PD98059 (0.1 μg/μl, EMD Biosciences, La Jolla, CA) dissolved in 10% DMSO, fluoroacetate (FA, 1 mM, Sigma-Aldrich, St. Louis, MI) or vehicle (isotonic saline). The pump was embedded subcutaneously in the dorsal portion of the body and then cervical spinal nerve was transected. Following recovery from the general anesthesia, PD98059, FA or vehicle was continually applied to the subdural space for 7 days (1 μl/h). The behavioral testing was conducted daily; there were no sign of any motor deficit.

### Effect of PD980859 or FA on ERK phosphorylation and astroglial cell and astroglial cell activation

The CNX and Sham rats (on Day 5 or 7 after the operation) with/without continuous i.t. administration of PD98059, FA or vehicle were applied low-intensity (6 g), medium-intensity (15 g) or high-intensity (60 g) mechanical stimulation by using von Frey filament (1 Hz; total duration of testing was 10 min) on lateral facial skin. Five min after the stimulation, the rats were perfused with 500 ml of 1% paraformaldehyde (PFA) in 0.1 M phosphate buffer (PB, PH 7.3) followed by 500 ml of 4% PFA in 0.1 M PB. The medulla and upper cervical spinal cord were removed and post-fixed in 4% PFA for 3 days at 4°C. The tissues were then transferred to 20% sucrose (W/P) in PBS for several days for cryoprotection.

### Immunohistochemistry

Fifty micrometer thick sections of Vc and upper cervical spinal cord were cut with a freezing microtome and every 8th section was collected in PBS. Free-floating tissue sections were rinsed in PBS and 10% normal goat serum (NGS) in PBS for 1 h, and then incubated in rabbit anti-phospho-p44/42 MAPK (Thr202/Tyr204) antibody (1:1000; Cell Signaling, Beverly, MA) for 72 h, rabbit anti-GFAP (1:1000; Dako Japan, Tokyo, Japan) for 3 days, rabbit anti-N-methyl-D-aspartic acid receptor 1 (NMDAR1) (phosphor Ser 896) antibody (1:150; Bioworld Technology, Minneapolis, MN) at 4°C. Next, the sections were incubated in biotinylated goat anti-rabbit IgG (1:600; Vector Laboratories, Burlingame, CA) for 2 h at room temperature. After rinsing, the sections were incubated in peroxidase-conjugated avidin-biotin complex (1:100; Vector Laboratories) for 1 h at room temperature. They were then washed in 0.05 M Tris buffer (TB), and next incubated in 0.035% 3.3'-diaminobenzidine-tetra HCl (DAB, Tokyo Chemical Industry, Tokyo, Japan), 0.2% nickel ammonium sulfate, and 0.05% peroxide in 0.05 M TB, pH 7.4. The sections were then washed in PBS, serially mounted on gelatin-coated slides, dehydrated in a series of alcohols (from 50 to 100%), and cover slipped. The pERK-like immunoreactive (LI) cells were drawn under a light microscope with an attached camera-lucida drawing tube (Neurolusida 2000, MicroBrightField, Colchester, UT). The number of pERK-LI cells in Vc and C1-C2 was counted from all sections, and the mean number of pERK-LI cells (per section per rat) was calculated from each animal.

GFAP is a specific marker of astroglial cells [12]. The area of the GFAP-labelled astroglial cells in Vc and C1-C2 was measured by using a computer-assisted imaging analysis system (Image J, National Institute of Health, Bethesda, MD). Three square boxes (200 × 200 μm^2^) were placed in the dorsal portion of the C2 dorsal horn (Figure 7Aa) and mean percent area occupied by anti-GFAP immuno-products was calculated in each rat.

The CNX or Sham rats on day 7 after operation were performed tissue preparation described above 5 min after receiving high-intensity (60 g) mechanical stimulation of the lateral facial skin (1 Hz, duration for 10 min). Free-floating tissue sections were rinsed in PBS and 10% NGS in PBS for 1 h, and then incubated in rabbit anti-phospho-p44/42 MAPK antibody (1:300) and mouse anti-NeuN antibody (1:1000; Chemicon, Temecula, CA) overnight at 4°C and secondary antibodies (anti-rabbit Alexa Fluor 488 IgG and anti-mouse Alexa Fluor 568, 1:200; Invitrogen, Carlsbad, CA) conjugated for 1 h at room temperature in a dark room. Then the sections were washed in PBS three times for 5 min, and mounted on slides and cover slipped in permaFluor (Sigma-Ardrich).

The CNX rats on day 5 after operation with i.t. administration of FA or vehicle were performed. Fifty micrometer thick sections of C1 were cut. Free-floating tissue sections were rinsed in PBS and 10% NGS in PBS for 1 h, and then incubated in rabbit anti-glutamine synthetase (GS, 1:5000; Abcam, Cambridge, UK) and mouse anti-GFAP (1:1000; Dako Japan) for 2 days at 4°C and secondary antibodies (anti-rabbit Alexa Fluor 488 IgG and anti-mouse Alexa Fluor 568, 1:1000; Invitrogen) conjugated for 2 h at room temperature in a dark room. Then the sections were washed in PBS three times for 5 min, and mounted on slides and cover slipped in permaFluor.

### Statistical analysis

The two-way analysis of variance (ANOVA) was performed on the behavioral test at each time point after the CNX with/without PD98059 and Sham operation. We also used one-way ANOVA on rank with post hoc Tukey or Dunnett's tests where appropriate. Differences were considered significant at p < 0.05.

## Competing interests

The authors declare that they have no competing interests.

## Authors' contributions

All authors read and approved the final manuscript. AK carried out the experiments and data analysis. SH, YT and AOO helped the experiments, data analysis and paper writing. BJS, KH and MS provided data interpretation and helped finalize the manuscript. YI and KI conceptualized the hypothesis, designed and supervised the experiments, directed the data analysis, and finalized the manuscript.

## Supplementary Material

Additional file 1 **Figure S1**. Photomicrographs of GFAP-labelled cells (A and D), GS-positive cells (B and E) and phosphorylated NR1-positive cells (G and H) in C2 in day-5 CNX (A-C) rats and those at day-5 Sham rats (D-F). C: merge A with B, F: merged D with E, G and H: NR1 positive cells in the CNX rats with i.t vehicle or FA administration, respectively. Arrows in G and H indicate NR1 positive cells. Click here for file
