# Supplementary figures and images for: Mechanisms involved in extraterritorial facial pain following cervical spinal nerve injury in rats
Source: Mol Pain. 2011 Feb 10;7:12. doi: 10.1186/1744-8069-7-12 (PMC3048571; doi:10.1186/1744-8069-7-12)

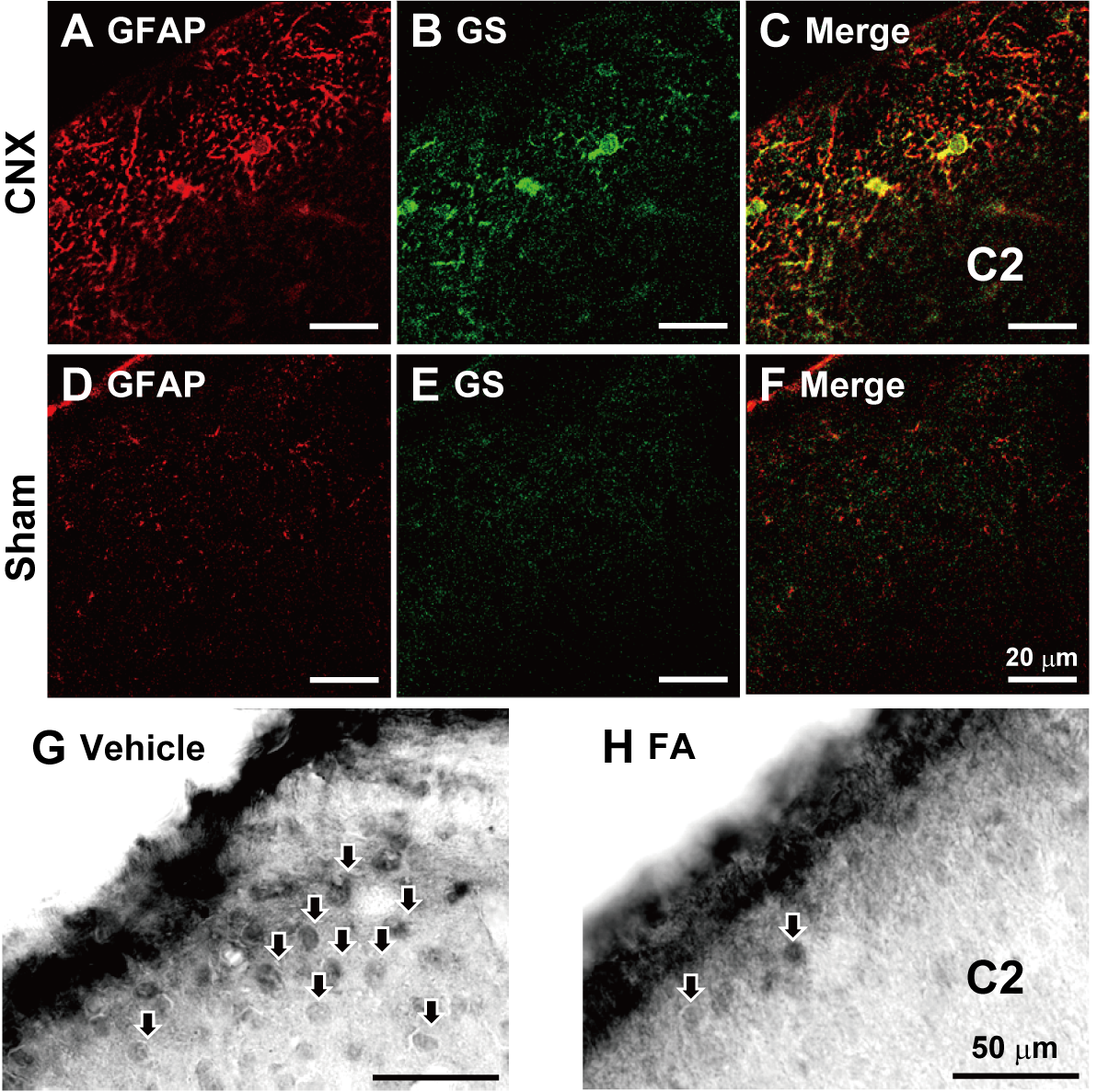

Supplement: Additional file 1 — Figure S1. Photomicrographs of GFAP-labelled cells (A and D), GS-positive cells (B and E) and phosphorylated NR1-positive cells (G and H) in C2 in day-5 CNX (A-C) rats and those at day-5 Sham rats (D-F). C: merge A with B, F: merged D with E, G and H: NR1 positive cells in the CNX rats with i.t vehicle or FA administration, respectively. Arrows in G and H indicate NR1 positive cells. [file 1744-8069-7-12-S1.PNG]
